# Comparing insecticide-treated nets access-use based on universal household and population indicators vis-a-vis measures adapted to sleeping spaces in Ethiopia

**DOI:** 10.1186/s12936-021-03887-9

**Published:** 2021-08-28

**Authors:** Yohannes Kebede, Morankar Sudhakar, Guda Alemayehu, Lakew Abebe, Zewdie Birhanu

**Affiliations:** 1 Department of Health, Behavior, and Society, Jimma University, Jimma, Oromia Ethiopia; 2 President’s Malaria Initiative, United States Agency for International Development, Addis Ababa, Ethiopia

## Abstract

**Background:**

Insecticide-treated nets (ITNs) access-use has been pivotal monitoring indicator for malaria prevention and control, particularly in resource limited settings. The objective of the study was to compare ITN access-use based on universal household and population indicators and measures adapted to sleeping spaces.

**Methods:**

A cross-sectional study was conducted in five districts of Jimma Zone, Ethiopia, March, 2019. 762 HHs were sampled for the survey. Multi-stage followed by simple random sampling used. Monitoring and evaluation reference group’s (MERG’s) indicators were used for measuring ITN access-use. MERG’s indicators are each adapted ITN access-use to sleeping spaces. Household (ownership, saturation and sufficiency) and population access and household members’ status of last night sleeping under ITN compared based on the two models. Differences of estimates of ITN access-use based on the two methods reported as magnitude of over/under estimations, at p-value < 0.05.

**Results:**

Based on MERG’s approach, the study revealed household (HH) based indicators as such: HH ownership of at least 1 ITN (92.6%), sufficiency of ITN for every two people in HH (50.3%), and saturation of ITN for every 2 people in HHs with any ITN (54.6%). Moreover, population based indicators were: population with ITN access (P3 = 78.6%), people who slept under ITN previous night (63.0%), people who slept under ITN among who accessed it (73.1%), ITN use-gap (26.9%). Equivalent indicators of HH ownership, sufficiency, saturation, and people accessed at where they actually slept, and people slept under ITN among those accessed at where they slept estimated at 71.3%, 49.4%, 69.3%, 66.3%, and 92.1%, respectively. MERG’s approach over-estimated ownership, people’s access, and behaviour-failures by 21.3%, 12.3%, 19.0%, respectively. Over-estimation occurred for reasons such as many sleeping spaces lack ITN and > 2 people actually slept per sleeping space.

**Conclusions:**

MERG’s universal indicators over estimated households and populations ITN access-use as a result of absence of measures capturing access-use values at spaces where people actually slept. Consequently, measures adapted to sleeping contexts revealed potential misdistributions practiced when the existing indicators are in use. Insertion of sleeping spaces into existing approach will be worthwhile and needs to be promoted as it improves curiosity in ITN distribution, produces closer estimates and prevents malaria prevention and control programmes from overlooking access-use challenges.

## Background

Despite significant achievements over the last decades, malaria remains an important public health problem [1–4]. Nineteen countries in sub-Saharan Africa and India carried almost 85% of the global burden of malaria [5, 6]. Nowadays, global malaria programme aspires to achieve malaria elimination by 2035 and global eradication by 2040–2050 [6–10]. Ethiopia has set national goal of eliminating malaria by 2020 (sub-national) and 2030 (national) [11, 12]. Ethiopian national malaria indicator survey (ENMIS) 2015 revealed that 65% of districts in the country were malarious [13]. Global technical strategy has been committed in rendering supports and commodities required to meet malaria prevention, control and elimination efforts in resource-limited settings [7]. In 2018, 197 million insecticide-treated nets (ITNs) were delivered globally, of which 87% were gone to sub-Saharan Africa [5].

ITN is one of the most effective malaria preventive tools especially in population at high risk [7–11]. Consequently, the Roll Back Malaria (RBM) recommends every member of household should sleep under ITN every night. The minimum population based utilization that could provide sufficient leverage to malaria prevention and control conventionally has been 80% [14–18]. Although behavioural factors were significant, many studies also indicated that access to ITN is a crucial factor [19–25]. Keeping other factors constant, there has been strong conviction that addressing access is presumptive to ITN use [14–18]. Ethiopian national strategic malaria prevention (NSMP) III plans to achieve 80% ITN use through 100% population access [11]. Given that access to nets has to precede usage, multiple approaches such as mass distribution campaigns, antenatal care facilities and immunization programmes were utilized to increase access in malaria-affected communities particularly in resource-limited settings [5, 26–28].

The RBM’s Household Survey Indicators (HIS) recommended seven indicators to measure proportions of household (HH) and population who have access to and use ITN [14–18]. Few studies were conducted based on the HSI estimates of HH and population coverage and use of ITN in resource limited settings [26–31]. At HH level, the HSI recommended the bottom-line access through ownership of at least 1 ITN per HH. Another HSI is sufficiency of the ITN in HHs owning at least 1, assuming that 1 ITN is sufficient for every two people in the HH [18, 32–34]. Mere presences of ITN in HHs do not necessary indicate individual access and use. Therefore, to further assure every person in the community utilizes ITN, RBM-HSI recommended indicators that could embrace access at population level beyond the proportion of HHs with ITN. This requires to be sure if all the ITNs available in the HHs were enough for every two persons in the HH. This is expected to meet the requirement that every member in a HH should use ITN at every night. When access is a challenge, pregnant women and children under-five years old should receive priority for sleeping under ITN [18, 33, 34].

Studies suggest people should access ITN at their own sleeping spaces (SS) and that was supposed to improve accuracy of ITN access-use [25–29, 35, 36]. NSMP-III also specifies access to ITN at sleeping spaces [11]. Sleeping space (SS), in this case, refers to any space or room that is arranged for sleeping in the surveyed HHs. Nonetheless, limited studies considered SS to understand determinants of ITN use [35, 36]. Therefore, the authors have strong conviction that the actual SS should be considered to enhance access-use of ITN at household level. This premises led to the need to compare ITN access-use based on existing HSI and those adapted to the context of SS. The authors intended to consider SS concurrent to the HSI, not to replace. To the best of the authors’ knowledge, limited studies focused on measuring access to ITN using contexts of sleeping spaces, and compared the estimates with the existing model of RBM. Therefore, it is appropriate to compare estimates of ITN access-use based on the HSI and the indicators adapted to SS, interpret the differences of the estimates, identify potential concurrence, and provide recommendation toward improved ITN access-use in resource-limited settings such as Ethiopia.

## Methods

### Study setting and period

The data were collected during March–April, 2019 from HHs in five districts of Jimma zone namely; Limmu-Kossa, Botor-Tolay, Gera, Shebe-Sombo and Nono-Benja. Jimma zone has been one of malaria endemic settings in Ethiopia. The districts are in the range of 70–229 km distance from Jimma town (the capital of the zone). They also have high to medium risk of malaria. Moreover, 20 gandas (the lowest administrative government structure of the study setting) were involved in the study from across the five districts. Across the districts, there are programmes rendering malaria prevention and control services and commodities such as ITN, IRS and treatments.

### Study design

The study used cross-sectional HH survey. The data was a portion of end-line assessment of a school-based social and behaviour change communication (SBCC) interventions which was aimed to advance community malaria preventive practices.

### Population and sample size

The survey was conducted among heads of the HHs. Given the study was nested with the SBCC project’s end line survey; the same sample (762 HHs) that was used for evaluating the project was taken. Details of assumptions used for calculating the sample size reported in separate published article [37].

### Sampling technique

Multi-stage sampling was used. First, the sample size was proportionally allocated to the study districts based on respective population sizes. Twenty gandas were randomly selected from across the five districts (4 from each district). The samples were allocated to each ganda according to their respective number of households. Finally, equal samples allocated to three administrative sub-divisions (called zoni) of the gandas. The HHs lists were obtained from family folders of the respective gandas. Then, the HHs were selected using computer generated random numbers. The selected HHs were traced through local guiders.

### Expected outcome variables

This study was mainly intended to examine access-use of ITN by target community based on HSI first, and then adapted with sleeping space. Access-use of ITN hypothesized to be more enhanced when ITN is accessed at actual sleeping space rather than mere availability in the HH even considering 1 ITN for every 2 people. Obviously, ITN prevents malaria when HH members access it at actual sleeping space. Therefore, how close the access-use of ITN based on HSI and SS was intended to be investigated.

### Measurements

Access-use of ITN was measured by standard tools adopted mainly from a 2018 RBM guidelines for household survey indicators (HIS) for malaria control [18]. The HSI include six HH and population based proportions (P1–P6). The same proportions were estimated after adjustment to sleeping space (SSP1–SSP6)-as alternative model of access-use.

#### Operational definitions

1. HSI based estimates: household and population coverage and use of ITN
  Indicator 1: Proportion of households (HHs) with at least one ITN (P1): measures HH ITN ownership i.e. the extent to which ITN programmes have reached all households or, conversely, the not yet reached.
  Indicator 2: Proportion of HHs with at least one ITN for every two people (P2): measures the proportion of HHs (all HHs surveyed as denominator) that have a sufficient number of ITNs to cover all individuals who spent the previous night in surveyed HHs, assuming each ITN is shared by two people. HHs with full coverage (enough nets) are indicated by value 1, the rest of HHs are assigned a value of 0.
  Indicator 3: Proportion of population with access to an ITN in their HH (P3): estimates the proportion of the HH population that could have slept under an ITN, assuming each ITN is used by two people. It is calculated from the total number of individuals, who could sleep under an ITN if each ITN in the HH were used by two people (potential ITN users) as numerator, and the total number of individuals who spent the previous night in surveyed HHs as denominator. A “potential ITN users” is an intermediate variable calculated by multiplying the number of ITN in each HHs by two.
  Indicator 4: Proportion of the population that slept under an ITN the previous night (P4): measures the level of ITN use among all individuals who spent the previous night in surveyed HHs, regardless of whether those individuals had access to an ITN in their HH.
  Indicator 5: Proportion of HHs with at least one ITN for every two people (P5) calculated from HHs with any ITN as denominator, indicating saturation of HHs with required number of ITN. Conversely, 1-P5 measures intra-household ITN ownership gaps that are HHs that own at least one ITN but do not have full coverage.
  Indicator 6: Proportion of the population that slept under an ITN the previous night (P6) calculated from individuals who had access to an ITN in their HH as denominator. Conversely, 1-P6 measures behavioural failures (potential users did not use ITN for reasons other than access).
2. Adapted to sleeping space (SS/SS) based estimates of ITN coverage and use
  In this case, sleeping space indicates any room or arrangement for sleeping in the HH. The above HSI were all adapted to where people sleep in the HHs.
  Indicator 1: Proportion of HHs with ITN in at least any one of the sleeping spaces in the HHs (SSP1): measures sleeping space ITN ownership i.e. the extent to which ITN programmes has reached SS in the HHs.
  Indicator 2: Proportion of HHs with ITN for every sleeping space in the HH (SSP2): measures the proportion of HHs having ITNs to cover all sleeping spaces in surveyed HHs, assuming each SS should have ITN.
  Indicator 3: Proportion of population with access to an ITN in their SS in the HH (SSP3): estimates the proportion of the people covered by ITN at their respective sleeping spaces in the HH. The calculation was based on the number of subjects who slept in the previous night in the household and had an ITN hanged in their sleeping space.
  Indicator 4: Proportion of the population that slept under an ITN the previous night (SSP4 = P4).
  Indicator 5: Proportion of HHs with ITN for every sleeping space (SSP5) calculated HHs with ITN in any of its SS as denominator. Conversely, the gap (1-SSP5) measures intra-sleeping space ownership gaps.
  Indicator 6: Proportion of the population that slept under an ITN the previous night (SSP6) calculated from people accessed with ITN at where they slept as denominator.

#### Explanations, relationships between indicators, and interpretations

P1, P2 and P5: In connection with P1, P2 can be used to determine what proportion of HHs already reached with at least one ITN have a sufficient number of ITNs to protect all members in the HH. P5 is an extension of P2, and indicates level of saturation of 1 ITN for every 2 people considering only HHs reached by any ITN (ITN distribution fairness). If the difference between these indicators is substantial, programmes need to assess whether current ITN distribution strategies should be revised to fill the saturation gap.

P3, P4 and P6: In HHs having more than one ITN for every two people, the potential users will be equal to the number of people who slept in that HH last night. Therefore, after dividing the sum of all potential ITN users in the sample by the total number of individuals who spent the previous night in surveyed HHs, P3 gives any value between 0 and 1 indicating the magnitude of people who access ITN (there could be occasions such as excess accumulation of ITNs i.e. greater than 1 ITN for every 2 people when the actual calculation of P3 yielded values > 1 but corrected to maximum value of 1 according to RBM’s definition). If the difference between P3 and proportion of population sleeping under ITN the previous night (P4) is substantial, the programme may need to focus on identifying the main drivers or barriers to ITN use to design an appropriate intervention (focus on achieving high ITN coverage or promotion of its use or both) for behaviour change. However, to be more specific as whether access or utilization behaviour intervention is required, P6 can best be considered as an extension of P4. This means the behaviour of sleeping under ITN last night of survey was limited to household members who had access to ITN (P3). Thus, substantial difference between P3 and P6 (at relatively large value of P3) indicates the magnitude of the behavioural gap in use of ITNs (i.e., the population that has access to an ITN but is not using it) informing that ITN programmes need to focus on promoting ITN use.

Indicators of SS: The above relationship and interpretation maintained except that P1/P2/P5 that considered ITN coverage and distribution to every two people at household level was interpreted based on every sleeping space in the households for SSP1, SSP2 and SSP5 (indicating the extent to which ITN can reach every SS of HHs in the community); and the last night’s potential users of available ITN used for P3, P4, and P6 was interpreted based on every human subject sharing sleeping spaces when ITN availed at where people actually slept (indicating ITN users should be accessed at where they actually slept).

### Data collection procedures

The data were collected through face-to-face interviewer administered method. The interview was conducted by trained experienced interviewers under supervision in Afaan Oromo language. Three days training about the purpose of the study, instruments and data collection procedures was given. The data were cleaned, checked for consistency on daily basis. The research team supervised overall data collection process.

### Data analysis

The data were analysed by using SPSS version 20.0. Operational definitions set by 2018 RBM’s HSI was referred and SS indicators was adapted to determine ownership-access-use of ITN. Differences of proportions were used with 95% confidence interval to describe the key indicators. Moreover, the discrepancies within and between was determined, the closeness of the estimates was compared, the implications was interpreted and finally relevance of complementation of the two HIS and adapted SS indicators was identified, particularly for ownership and access measures. Moreover, a diagrammatic flowchart of ITN distribution and coverage operational algorithm for improved access-use of ITN proposed to malaria prevention and control programmes.

### Ethical approval and considerations

The study was approved by Jimma University, Institute of health institutional review board for Institute of Health. Official permissions to undertake the study were obtained from concerned bodies. Respondents were given detailed information about the purpose of the study. Informed written consent was obtained from all study participants.

## Results

### Socio-demographic characteristics of respondents

In this study, a total sample of 759 HHs and 3760 people participated. The response rate was 99.6%. Average age of the respondents was 36.9 ± 13.3 years old. Average family size per HH was 4.9 ± 2.2 members. Table 1 contains detailed background information of the respondents.

**Table 1:** Socio-demographic characteristics of household respondents, Jimma Zone, Ethiopia, March, 2019 (n = 759 HHs)

Socio-demographic characteristics	Descriptive statistics	
	No	%
Sampled people (Districts)		
Botor-Tolay [HH = 120]	606	16.1
Nono-Benja [HH = 123]	651	17.3
Limmu-Kossa [HH = 132]	615	16.4
Gera [HH = 166]	759	20.2
Shebe-Sombo [HH = 218]	1129	30.0
Total HH = 759	3760	100
Sex of respondent		
Male	439	57.9
Female	320	42.1
Sex of HH head		
Male	690	90.9
Female	69	9.1
Marital status		
Married	642	85.0
Divorced	31	4.1
Widowed	29	3.8
^a^Others	53	7.2
Education		
No read nor write	313	41.2
Read and write	80	10.6
Primary school	251	33.1
Secondary school	106	14.0
College and above	5	1.1
Religion		
Muslim	423	55.9
Orthodox	230	30.4
Protestant	104	13.7
Ethnicity		
Oromo	576	76.0
Amhara	138	18.2
^b^Others	54	7.0
Proportions of		
< 5 years old population	584	15.5
School age children (5–14)	835	22.2
Adults per HHs	2343	62.3
PW/15–49 years (females)	31	3.4
Average family size	4.9 (mean)	2.2 (SD)

*HH* household, *PW* pregnant women, *SD* standard deviation

^a^Others: single, cohabitation, ^b^Others: Gurage, Tigre, Dawro, Kaffa, Hadiya, and Kambata

### Description of sleeping spaces and their relevance for accessing people with ITN

Table 2 provides the details of sleeping spaces and ITN. The median sleeping spaces in the HHs was found to be 2. Averagely, 2.5 people slept in any sleeping space across HHs. In 75% of the sleeping spaces, there existed more than two persons slept in aggregate. People who slept in 6.6% of the spaces were the same sex. The probability of getting high-risk group at any sleeping space was 55% for < 5 children and 4.2% for pregnant women. The ratio of 1 ITN for median number of aggregates of persons per sleeping space (2.5) to RBM’s access conversion factor (1 ITN for every 2 people) was 0.8, labelled as SS relevance factor. This means 80% of ITN accessed one for every two people in HHs would be enough to cover same population if it is accessed at where people actually sleep.

**Table 2:** Description of sleeping spaces and people’s availability in it, Jimma Zone, March 2019, Ethiopia (N = 762 HHs)

Variables	Descriptive statistics	Estimates
Number of sleeping spaces in the HH	1	219 (28.7%)
	2	365 (47.9%)
	3	160 (21.0%)
	4	18 (2.4%)
	Mean	1.97
	Median	2.0
Aggregates of people per space		
Average measures	Mean	2.5
	Median	2.5
Quartiles	1st	2.0
	2rd	2.5
	3rd	3.0
Priority population’s covered by ITN at where space they slept		
Pregnant women	Mean	4.2 (3.4–5.2)
Under five children	Mean	55 (45–66)
Sex segregation per sleeping space	Same	50 (6.6%)
	Mixed	712 (93.4%)
Relevance factor of space-based access		
Median people actually accessed 1 ITN/SS	Space-based	1/2.5
Median people expected to access 1 ITN/2 people	RBM’s-based	½
Relevance factor for space-based measure	Space/RBM	0.8

### Availability and current status of ITN

Table 3 provides the details of ITN status. Each HH owned 2.1 ITNs, on average. The mean age of the ITNs was 13.6 months. In more than half of the HHs with any ITN, 404 (53.3%), at least 1 ITN was kept folded/saved in cabinet. In those HHs, 672 (45.6%) of all available ITNs were folded/saved. 186 (24.6%) of HHs had any ITN with repairable damage. About 252 (17.1%) of ITNs were washed across the observed HHs. There were 37 (2.5%) potentially functional ITNs reported as used for unintended purposes: some put under beds to kill insects other than malaria mosquitoes, rope, sacks, among others.

**Table 3:** Characteristics and perceptions about ITN and sleeping spaces in HHs, Jimma Zone, Ethiopia, March 2019 (N = 762 HHs)

Variables	Estimates	95% CI
ITN-characteristics		
Total number of functional ITNs in all HHs	1600	
Average number of ITN (HH) (mean)	2.1	2.0, 2.2
Age of ITN (in months)—mean	13.6	13.2, 14.1
ITN observability rate	1475 (92.2)	90.2, 94.3
Observed status of ITN		
HHs as denominator (N = 758)		
Any ITN tied on bed	575 (75.9)	72.9, 78.9
Any ITN kept folded/saved	404 (53.3)	49.9, 57.0
^a^Any ITN misused—other purpose	37 (4.9)	3.3, 6.7
ITN as denominator (N = 1475)		
Tied on the bed	766 (51.9)	47.7, 56.9
Kept folded and saved	672 (45.6)	39.9, 51.3
^a^Misused—other purpose	37 (2.5)	2.3, 2.7
ITN care status		
HHs as denominator (N = 758)		
Any ITN damaged (has hole)	186 (24.6)	21.5, 27.8
Any ITN washed (with last 3 months)	123 (16.3)	13.6, 18.9
ITN as denominator (N = 1475)		
Damaged (has hole) rate	246 (16.6)	13.5, 17.8
ITN wash rate (with last 3 months)	252 (17.1)	13.4, 18.9

^a^Other purposes-used as sheet beneath bed, stored cereals in, and shield on toilet

^b^Functional ITN: ITNs that can be utilized for sleeping under it, at least after minimal amendments

### Household and population access and use of ITN: HSI and SS models

#### Based on HSI model (existing)

Table 4 presents discrepancies household and population-based indicators between the two models. Proportion of HH ownership (P1), sufficiency (P2) and saturation (P5) of ITNs were 92.6%, 50.3%, and 54.6% respectively. On the other hand, corresponding magnitudes of people who accessed and slept under ITN the previous night were 78.6% (P3) and 63.0% (P4). The use-gap among people with access was 26.9% (1-P6).

**Table 4:** Comparison of HH and population’s ownership-access-use of ITN based on HSI and SS approaches, Jimma Zone, March 2019 (N = 762 HHs)

ITN ownership, access and use measures	Existing (1)	Concurrent (2)	Comparison (1–2)
	HIS	Adjusted (SS)	Discrepancy
Household based indicators			
Estimates (HSI, Sleeping space)	% (95% CI)	% (95% CI)	% (95% CI)
Ownership (P1, SSP1)	92.6 (90.1, 94.1)	71.3 (67.7, 74.4)	+ 21.3 (15.6, 26.3)*
Ownership gap (1-P1, 1-SSP1)	7.4 (6.0, 10.0)	28.7 (25.6, 32.3)	− 21.3 (− 26.3, − 15.6)*
Sufficiency (P2, SSP2)	50.3 (46.5, 53.6)	49.4 (45.5, 53.0)	+ 0.9 (− 7.5, 8.1)
Saturation (P5, SSP5)	54.6 (51.0, 58.1)	69.3 (67.2, 71.2)	− 14.7 (− 16.7, − 13.1)*
Intra-household gaps (1-P5, 1-SSP5)	45.4 (41.9, 49.0)	30.7 (28.8, 32.3)	+ 14.7 (13.1, 16.7)*
Population based indicators			
Population access within-HH (P3, SSP3)	78.6 (74.6, 81.7)	66.3 (62.3, 70.5)	+ 12.3 (4.1, 19.4)*
Access gap (1-P3, 1-SSP3)	21.4 (2.3, 10.4)	33.6 (36.3, 42.9)	− 12.3 (− 19.4, − 4.1)*
Slept under ITN previous night (P4 = SSP4)	63.0 (61.0, 64.0)	63.0 (61.0, 64.0)	Not calculated
Slept under ITN among accessed (P6, SSP6)	73.1 (68.5–77.5)	92.1 (89.2, 94.6)	− 19.0 (− 26.1, − 11.7c)*
Use-gap/behavior failure (1-P6, 1-SSP6)	26.9 (22.5, 31.5)	7.9 (5.4, 10.8)	+ 19.0 (11.7, 26.1)*
Use-access ratio-HH (P4/P3, SSP4/SSP3)	80.2 (78.3, 81.8)	95.0 (90.8, 97.9)	− 14.8 (− 19.6, − 9.0)*

*P-value < 0.05

#### Based on SS model (concurrent)

Proportions of ownership (SSP1), sufficiency (SSP2) and saturation (SSP5) of ITNs adjusted to the sleeping spaces in the HHs were 71.3%, 49.4% and 69.3%, respectively. People who accessed ITN where they actually slept in the HH was (SSP3 = 66.3%), people slept under ITN among those accessed where they slept (P6 = 92.1%). The ITN use-gap among people with access at sleeping space was 7.9% (1-SSP6) (Table 4).

### Discrepancies between the models

The two models produced statistically significant differences on access-use indicators except sufficiency. The HSI approach over-estimated some indicators. For example, population access (by 12.3%) and ownership (by 21.3%) compared to the SS approach. Nonetheless, the SS model under-estimated the magnitude of people who slept under ITN among who accessed (by 19.0%) and saturation (14.7%). Inversely, it over-inflated intra-HH gaps and behavioural failures. Overall, the use to access ratio marked the highest discrimination between the models as the ratio adjusted with SS produced 27.5% excess odds of ITN use (Table 4).

## Discussion

This study generated pertinent findings related to operational challenges of measuring ITN access-use in resource-limited settings. Obviously, numerous programmes are committed to provide ITN support as malaria preventive commodity to countries endeavouring to meet elimination plans [7–10]. This study explored and recommended introduction of new supportive indicator into RBM’s existing ones in order to improve access-use of ITN. For example, this study has found out HSI estimates of ITN access-use were relevant for detecting mis-distribution even though they over or under estimated some indicators compared to when adjusted with SS. Thereof, the complementation of the HSI with SS was discussed based on ITN-access indicators.

According to this study, the HSI approach indicated high HH ownership of ITN (P1) i.e. proportion of HHs with at least 1 ITN = 92.6%. EMIS 2015 reported 63% ownership in rural malarious settings [13]. Many studies and evaluation of mass distribution programmes revealed ownership to be high compared to other access indicators [26–30]. The EMIS 2015 estimates national ownership at 63.7%, the lowest being 33.9% in Dire Dawa and the highest being 72.9 in Amhara and Tigray regions each [13]. On the contrary, the SS adjusted ownership indicator showed moderate amount (SSP1 = 71.3%), i.e. availability of ITN in any SS in the HH. Noticeably, this huge gap between the existing and adapted model could be attributed to the inherent assumptions: the later defines ITN ownership should be based on spaces where people actually sleep not just the HH that can potentially have several sleeping arrangements (P1 compares HHs with sleeping space in the HH). Clearly, ITN use behaviour in itself is about “sleeping under it every night” i.e. impossible to use ITN unless actual sleeping spaces are considered. Ethiopian NMSP-III clearly strategized 100% sleeping spaces should own ITN [11]. Therefore, the HSI indicator (P1) overestimated ownership/underestimated ownership-gap by 21.3%, indicating significant number of sleeping spaces that were considered ‘owned ITN’ did not actually own because people did not own them in spaces where they actually slept. Estimations based on HHs do not necessarily ensure people are sleeping under ITN. The study showed the HSI indicator-HHs with sufficient ITN i.e. proportion of HHs with at least 1 ITN for every 2 people in the HH, P2 = 50.3%. EMIS 2015 reported 33% ownership in rural malarious settings [13]. Many studies showed closer estimate for HH ITN sufficiency [29, 30]. Nonetheless, low magnitude of sufficiency at large estimate of ownership signifies abnormal allocation of ITN (discrepancy = 42.3%HHs), either due to supply shortage, deprivation or unreported over concentration of ITN in certain HHs (there is ignorance of potential excess presence of ITN inherent to calculating P2) [32–34]. On the other hand, equivalent indicator of SS approach quantified a very close estimate of ITN sufficiency (SSP2 = 49.4%) for aggregate people in every sleeping spaces in the HHs. In resource-limited settings many people share living rooms [35]. EMIS 2015 reported 1.7 mean number of sleeping spaces [13]. Certainly, sleeping space is logical way to explore access worthy of consideration during ITN distribution.

In this study, saturation of ITN i.e. proportion of HHs with at least 1 ITN for every 2 people in HHs with any ITN was, P5 = 54.6%. EMIS 2015 reported national estimate of ITN saturation at 31.7% (lowest, 13.8% in Harari, and highest, 41.4% in Tigray) [13]. When compared to high ownership (P1 = 92.6%), this figure explains unfairness committed during ITN distribution in that 38.0% of all HHs claimed to own ITN did not have enough for every two people, causing substantial intra-household gaps (1-P5 = 45.4%). Nonetheless, SS based equivalent (SSP5 = 69.3%), yielded in sensible variation. Therefore, the HSI P5 underestimated saturation of ITN/overestimated intra-household gaps by 14.7% for couple of reasons: (1) the SS approach produced higher magnitude of saturation because more than 2 aggregate of people slept together in a given space in the HH, while HSI P5 utilizes conversion factor of 1 ITN for 2 people: indicating fewer number of ITNs are expected to meet saturation, (2) conceptually, 1-SSP5 underscores the question of accessing ITN at sleeping spaces while 1-P5 could unnecessarily report intra-HH gaps of accessing ITN in community wherein excess presence of ITN in some HHs could be observed (in fact, the formula hides this occasion) [32–34]. This means an emphasis to sleeping spaces during distribution of ITN can improve HHs saturation by increasing curiosity to people at where they sleep in the HH.

Based on the HSI approach this study produced population access, P3 = 78.6% i.e. proportion of people in the HHs who accessed 1 ITN for every 2 people. It meant that, 78.6% of populations in study districts were potential users of ITN during last night of survey. This estimate is closer to previous similar studies [25–30]. The figure did not yet reach the national target of 100% people’s access to ITN [11]. This indicated that ITN programme should still focus on coverage [8]. Nonetheless, in order to show key problems inherent to the calculation of P3 (based on operational definition given by RBM), we also calculated P3 without correcting higher access values (> 1) to 1. For the sake of discussion we named this indicator, uncorrected P3 (we introduced about the correction involved during P3 calculation in methods, sub-section mentioning about explanation, relationship between indicators and interpretations). Not correcting higher access values (> 1) to 1 can indicate several ITN programme implementation challenges such as misdistribution, over-accumulation in certain HHs or areas, and reasons behind those actions. Accordingly, the uncorrected P3 yielded 93.6%, the quantity that obviously looks overblown by 15% compared to the corrected P3 (more simply, P3 reported earlier by this study). Studies that did not adjust (correct) excess presence/accumulation of ITN in some HHs during calculation yielded lesser values of P3 than they could have estimated had the distribution strategies and systems been carefully operated to reach people in every malarious area and HH. This meant that using the P3 (while there are too many values > 1) encourages over-access in some HHs and indirectly supports misdistribution of ITN. This would be a critical challenge to ITN programme, particularly in settings, where ITN sufficiency and saturation values are observed to be low, and the case similar to the current study [30].

High magnitude of uncorrected P3 (= 93.6%) at high HH ownership (P1 = 92.6%) and low sufficiency (P2 = 50.3%) and saturation (P5 = 54.6%) implies: (1) excess accumulation of ITN in significant number of HHs; (2) inadequate availability of ITN in HHs with any ITN; (3) the ITN’s accumulated in those HHs was as high as that could have covered the overall community’s access challenge (P3–P5), that is 39% of peoples in HHs with any ITN were claimed to have access while actually not; (4) the P3 evenly distributes both potential accumulations in certain areas/HHs and inadequacy of ITN, that in turn potentially misclassifies access status.

The SS approach’s equivalent indicator (SSP3) revealed 66.3% i.e. proportion of people who accessed ITN in aggregates at their sleeping spaces. Ethiopian national 2020 target of 100% people’s access to ITN in malarious areas clearly mentioned access at where people actually slept [11]. Compared to P3, the SSP3 produced more reliable estimate of population access. The comparison of SSP3 and P3 revealed the failure of the P3 to pick excess presence by 27.3% (when P3 is uncorrected P3) and even 12.3% (when P3 is corrected). This means that about 27.3% (uncorrected P3) or 12.3% (P3) of people who never accessed ITN at their sleeping spaces were claimed to have access to 1 ITN for two (i.e. P3-SSP3) in the HHs they have slept last night. The ITN saving and misuse status reported in the current study also supports the idea that the corrected P3 leads to wrong estimation of access in settings with limited resource and system of distribution. For example, 48.1% of ITNs available across the HHs were saved or misused. Excess presence of ITN in the HHs was one of the potential factors for saving or misuse [37]. Many studies report mis or multipurpose use of ITN [21–26, 35, 36, 38].

This study observed 63.0% of people slept under ITN previous night of the survey. Many studies reported closer estimate [21–24, 35, 36]. World malaria report 2019 revealed ITN use was 50–80% in sub-Saharan Africa [5]. Moreover, this amount of ITN use was less than expected compared to national target by 2020, that was, 80% [11]. The finding from this study showed proportion of people who slept under ITN among those who accessed (P6) = 73.1% indicating 26.9% use gaps. This designated the amount of people who failed to use ITN for reasons other than access. This revealed that ITN programme should also focus on promoting ITN utilization behaviour through different strategies. School-centered community education strategy promoted ITN utilization [37, 39]. Concurrently, the SS equivalent (SSP6) was 92.1% producing only 7.9% behavioural failure to use ITN. Myriads of studies reported ethnic minority, income, house types, bed net compatibility to the sleeping spaces, types of roofs, risk perception and response-efficacy of bed nets, and descriptive norms, among others were potential reasons/factors of the behavioural failures [40–43]. In fact, some of the reasons such as roof/house types reported in these studies as behavioural failure were still structural incompatibilities; needing redefining what access meant in the light of sleeping arrangements of expectant users. In support of this thought, the discrepancy (26.9–7.9%) of 19% in estimates of behaviour failure explained by the HIS—over-estimated use of ITN among accessed ones by 19%. This implied: people tend to use ITN better when they were accessed at their sleeping space than do just allocating 1 ITN for every 2 persons in the HH. Moreover, when ITNs are fixed at sleeping spaces it involves sense of feeling accessed, prevents misuse and unnecessary accumulation. Studies and national plans show accessing ITN at sleeping spaces worthwhile [11, 29, 35].

According to this study, the relevance factor of sleeping spaces was 0.8 of the HSI. This means accessing ITN based on where people actually sleep can save 20% of ITN accessed to people through existing formula of 1 ITN per two people in HHs. For example, a total of 1600 ITNs counted across the sampled HHs in this study resulted in 78.6% population access. When adjusted to the SS, the same indicator will be 0.8 * 1600 = 1280, indicate 320 extra ITNs remain after covering 78.6% people at where they actually slept. In other words, given 2.5 average number of people slept in any sleeping space, in the current study, the 1600 ITNs would have served 98.3% people if properly distributed to people by considering where people actually sleep in the HH. This ratio only indicated relevance in terms of quantity of ITN, not the quality of size and form of its production. The relevance of approaching access to ITN based on sleeping space, concurrent to the RBM’s existing approach, can support policy makers and implementers and signals curiosity during ITN distribution and even manufacturing, particularly for resource-limited settings.

**Fig. 1:**
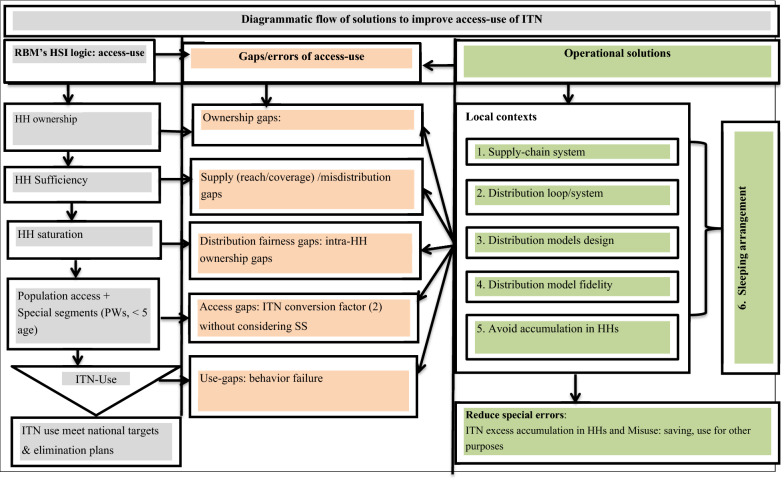
Diagram for operational framework for access-use of ITN involving sleeping space in resource limited setting, March 2019

Overall, this study was the first of its kind to compare SS with HSI approaches. In fact, some studies from African countries such as Mozambique and Rwanda commented on the need to consider sleeping spaces to measuring access [44, 45]. However, the study was not without limitation. The findings were not well discussed with other literatures and studies, given similar studies were limited. Furthermore, the study did not address about suitability of the ITNs’ structural design and maps of sleeping spaces. In general, the study explored considerable discrepancy between the HSI and adjusted approaches in estimating access-use-gaps of ITN. Thereof, framework that is believed to correct the distribution-access-use gaps of ITN in the study settings was designed as presented in the following diagram by Fig. 1. The EMIS 2015 plainly reported about huge mismatches between coverage of ITN and administrative reports and observed gaps regarding the ITN distribution management. For example, the EMIS reported that the household level coverage of LLINs was 64 percent despite FMOH claim of delivery to almost all malarious districts visited. Still, the EMSI reported that there were undistributed ITNs in several district offices [13].

## Conclusions

HSI model underlined the existence of ITN misdistribution: unfairness and unacceptable excess presence in certain HHs while there was unsaturation in significant proportion of HHs at the same time. HIS also under estimated access-use gaps, and evenly distributed over accumulations and insufficiencies of ITN in community where overall access gaps were observed although ITN utilization should be individuals practice at every night. Insertion of sleeping space into existing ITN programme strategies will be worthwhile and should be promoted; as it improves curiosity in ITN distribution, produces closer estimates of access-use indicators, and prevents from overlooking implementation of access-use challenges. Overall, local malaria prevention and control programme is recommended to consider the RBM’s existing HSI of ITN access-use with adjustment to sleeping spaces.

## Data Availability

All relevant data are within the manuscript and its Additional files.

## Abbreviations

AIM: Action and Investment to Defeat Malaria
GTS: Global technical strategy for malaria
IRS: Indoor Residual Spraying
ITN: Insecticide-treated net
ENMCP: Ethiopian National Malaria Control Programme
SBCC: Social and Behavioural Change Communication
PMI: President’s Malaria Initiative
RBM: Roll Back Malaria
WHO: World Health Organization
